# Polydatin and Resveratrol Inhibit the Inflammatory Process Induced by Urate and Pyrophosphate Crystals in THP-1 Cells

**DOI:** 10.3390/foods8110560

**Published:** 2019-11-07

**Authors:** Francesca Oliviero, Yessica Zamudio-Cuevas, Elisa Belluzzi, Lisa Andretto, Anna Scanu, Marta Favero, Roberta Ramonda, Giampietro Ravagnan, Alberto López-Reyes, Paolo Spinella, Leonardo Punzi

**Affiliations:** 1 Rheumatology Unit, Department of Medicine—DIMED, University of Padova, 35128 Padova, Italy; yesszamudio@gmail.com (Y.Z.-C.); elisa.belluzzi@gmail.com (E.B.); lilli.1991@hotmail.it (L.A.); anna.scanu@unipd.it (A.S.); faveromarta@gmail.com (M.F.); roberta.ramonda@unipd.it (R.R.); punzileonardo@gmail.com (L.P.); 2 Laboratorio de Líquido Sinovial, Instituto Nacional de Rehabilitación “Luis Guillermo Ibarra”, 14380 Mexico City, Mexico; allorey@yahoo.com; 3 Institute of Translational Pharmacology—National Research Council, 00185 Rome, Italy; gprav@unive.it; 4 Clinical Nutrition Unit, Department of Medicine—DIMED, University of Padova, 35128 Padova, ltaly; paolo.spinella@unipd.it; 5 Centre for Gout and Metabolic Bone and Joint Diseases, Rheumatology, SS Giovanni and Paolo Hospital, 30122 Venice, Italy

**Keywords:** polydatin, resveratrol, urate crystals, pyrophosphate crystals, crystal-induced inflammation

## Abstract

Resveratol (RES) and its natural precursor polydatin (PD) are polyphenols that may display a broad variety of beneficial effects including anti-inflammatory properties. This study aimed to investigate the role of RES and PD in the inflammatory process induced by monosodium urate (MSU) and calcium pyrophosphate (CPP) crystals in vitro. A monocytic cell line (THP-1) was primed for 3 hours with phorbol myristate acetate (100 ng/mL) and stimulated with synthetic MSU (0.05 mg/mL) and CPP (0.025 mg/mL) crystals. RES and PD were added to cultures concurrently with the crystals, or as 2-hour pretreatment. The effect of the two polyphenols was evaluated on intracellular and extracellular IL-1β levels, NACHT-LRRPYD-containing protein-3 (NLRP3) inflammasome expression, reactive oxygen species (ROS) and nitric oxide (NO) production, and the assessment of crystal phagocytosis. RES and PD strongly inhibited IL-1β induced by crystals after cell pretreatment. Cell pretreatment was effective also in reducing IL-1 mRNA expression while no effect was observed on NLRP3 gene expression. RES and PD had no effect on crystal phagocytosis when used as pretreatment. Both polyphenols were significantly effective in inhibiting ROS and NO production. Our results demonstrated that RES and PD are effective in inhibiting crystal-induced inflammation. Data obtained after cell pretreatment allow us to hypothesize that these polyphenols act on specific signaling pathways, preventing inflammation.

## 1. Introduction

The deposition of pathogenic crystals, such as calcium pyrophosphate (CPP) and monosodium urate (MSU) crystals in joints, triggers an important inflammatory reaction which leads to swelling, pain, limited joint function and, over time, tissue damage and disability. This process, which is characterized by an abnormal production of chemokines, cytokines, and reactive oxygen species (ROS) by the inflammatory cell infiltrate, can lead to chronic arthritis, namely, crystal-induced arthritis [1,2]. Since the time cytoplasmic NACHT-LRRPYD-containing protein-3 (NLRP3) inflammasome was first identified and its activation by MSU and CPP crystals was demonstrated [3], IL-1β has been considered the most important inflammatory mediator in crystal-induced inflammation, and it represents one of the main targets for new therapeutic approaches that have been or are being developed to treat crystal-related diseases [4,5,6].

Currently, corticosteroids, non-steroidal anti-inflammatory drugs (NSAIDs), and colchicine are effective drugs used to treat the acute attack in both gout, caused by MSU crystals, and pyrophosphate-related arthropathies, caused by CPP crystals [7]. However, while MSU crystal deposition can be prevented by treating hyperuricemia with urate-lowering therapy, there are no drugs able to prevent CPP crystal formation [6]. 

In recent years, significant interest has emerged in the beneficial health effects attributed to polyphenols. These plant-derived natural compounds have demonstrated a wide range of anti-inflammatory, anti-oxidant, and immunomodulant effects in many inflammatory chronic conditions [8,9,10,11]. 

Few years ago, we showed that the green tea polyphenol epigallocatechin-gallate affects the inflammatory response to CPP crystals by inhibiting chemokine release, chemotaxis, and interfering with membrane cell organization [12]. In the present study, we investigated the effect of a typical polyphenol of the Mediterranean diet, resveratrol (trans-3,5,4-trihydroxystilbene, RES) and its natural precursor, polydatin (3,5,4-trihydroxystilbene-3-O-beta-D-glucopyranoside, PD) in the in vitro model of crystal-induced inflammation. These compounds are mainly found in the roots of *Polygonum Cuspidatum*, grape, and red wine, and have shown a wide range of anti-inflammatory, anti-oxidant, and anti-tumoral properties [13,14,15,16].

However, only a few studies have evaluated the role of RES and PD in crystal-induced inflammation. While RES has shown to have an antihyperuricemic effect in mice [17,18] and to suppress inflammation through the inhibition of NLRP-3 inflammasome assembly [19], no studies have assessed the role of these polyphenols in the inflammatory process induced by CPP crystals.

We therefore assessed the influence of RES and PD on the IL-1β pathway and oxidative stress induced by crystals in THP-1 cells, suggesting an important role of these substances in preventing crystal-induced inflammation.

## 2. Materials and Methods

### 2.1. Reagents

THP-1 cells were purchased from the American Type Culture Collection^®^. CPP and MSU crystals were from InVivo Gen, Toulouse, France; phorbol myristate acetate (PMA), RPMI 1640, fetal calf serum, polymyxin B and phosphate buffer saline (PBS) were from Sigma-Aldrich, Milan, Italy.

### 2.2. Polyphenols

PD was extracted from *Polygonum Cuspidatum*, according to the procedure described in patent EP 1292320 B1 and kindly supplied by GLURES Srl (a spin-off of the National Research Council, Rome, Italy, purity >99%). RES was purchased from Sigma-Aldrich. 

Both the compounds were dissolved in ethanol at 100 mM stock solution and diluted in culture medium prior to use.

### 2.3. Cell Culture and Treatment

THP-1 cells were cultured in RPMI 1640 medium supplemented with 10% heat inactivated fetal calf serum. Cells were primed for 3 h with PMA at 100 ng/mL and resuspended overnight with fresh medium supplemented with 10% fetal calf serum.

The following day, the cells were plated at a density of 50.000 cells/0.32 cm^2^ and stimulated with sterile CPP or MSU crystals (cell culture tested and unable to activate TLR4-expressing cells) at the final concentration 0.025 and 0.05 mg/mL, respectively, and cultured for 24 h in presence of PD or RES [12]. Preliminarily dose–response experiments using different polyphenol concentrations ranging from 0.1 to 200 µM were conducted to determine the optimal RES (100 µM) and PD (200 µM) experimental concentrations.

Additional experiments have been performed after a 2 h pretreatment with PD and RES followed by their complete removal from the medium and subsequent stimulation with the crystals. Cells incubated with medium and polyphenols alone served as controls.

To exclude any contribution by endotoxin contamination, 10 mg/mL of polymyxin B was included in all the stimulation assays. All the experiments were performed three independent times.

### 2.4. Cell Viability, Death and Apoptosis Assays

Cell viability and proliferation were evaluated using the colorimetric MTT (3-(4,5-dimethylthiazol-2-yl)-2,5-diphenyltetrazolium bromide) mitochondrial activity assay (Chemicon) [20]. Cells were cultured in a 96-well plate and exposed to crystals, PD and RES for 24 h. 

After removing the supernatant, cells were treated with the MTT solution according to the manufacturer’s procedures and the absorbance intensity measured at 570 nm. The cell viability (%) was expressed as the percentage of cell survival in each group relative to the untreated control cells. 

Cell death and apoptosis were assessed by image-based cytometry using the Alexa Fluor^®^ 488 Annexin V/Dead Cell Apoptosis Kit (Molecular Probes).

### 2.5. IL-1β Measurement

Cell culture supernatants from different experimental conditions were examined for extracellular IL-1β concentration using Thermo Fischer Scientific ELISA kits (Waltham, MA, USA, detection limit 4 pg/mL). Intracellular IL-1β was determined in lysates obtained after three freeze–thaw cycles and resuspended in PBS [21].

### 2.6. Gene Expression Analysis

RNA extraction was carried out with the RNeasy mini kit (Qiagen, Milano, Italy) according to the manufacturer’s instructions. RNA was treated with DNase (DNA-free kit, AMBION, Thermo Fisher Scientific, Waltham, MA, USA) to remove possible contaminating DNA and then reverse-transcribed using using a iScript™ cDNA Synthesis Kit (Bio-Rad, Milano, Italy) according to the manufacturer’s instructions [22]. Quantitative real-time PCR (qPCR) was then carried out on a CFX96 Real-Time instrument (Bio-Rad), using Sso Fast Eva Green Supermix (Bio-Rad) according to the manufacturer’s instructions. Levels of mRNA for each target gene were normalized to glyceraldehyde-3 phosphate dehydrogenase (GAPDH) and calculated according to the 2-ΔΔCt method [23]. The sequences of primers are reported in Table 1.

### 2.7. Assessment of Intracellular Oxidative Stress

Oxidative stress was evaluated through the determination of intracellular ROS by CellROX^®^ Deep Red Reagent, a cell-permeable fluorogenic probe which exhibits excitation/emission at 640/665 nm upon oxidation [20]. The reagent was added at a concentration of 5 µM to the cells stimulated with crystals, RES and PD for the concentrations and indicated time, and incubated for 30 min at 37 °C. 

The medium was then removed, and the cells washed three times with PBS. The fluorescence was measured using a fluorescence microscope (EVOS; FL Auto Cell Imaging System, Thermo Fisher, Waltham, MA, USA) and quantified using a Tali Image-based Cytometer (Thermo Fisher, Waltham, MA, USA) according to the manufacturer’s instructions. Data analysis was performed based on fluorescence intensity expressed as arbitrary fluorescence units (AFU).

### 2.8. Nitric Oxide Determination

Intracellular NO was quantified using the commercial kit DAF-FM (4-amino-5-methylamino-2,7-difluorofluorescein diacetate, Molecular Probe), a pH-insensitive fluorescent dye that emits increased fluorescence after reaction with an active intermediate of NO formed during the spontaneous oxidation of NO to NO_2_- at 515 nm. The fluorescence was quantified using a Tali Image-based Cytometer (Thermo Fisher, Waltham, MA, USA), according to the manufacturer’s instructions. Data analysis was performed based on fluorescence intensities expressed as AFU.

### 2.9. Evaluation of CPP and MSU Crystal Phagocytosis

The phagocytosis of CPP and MSU crystals was assessed at different time points in the presence of RES and PD. Intracellular crystals were evaluated by means of ordinary and polarized light microscopy. The percentage of cells with internalized crystals was calculated on the total number of examined cells and expressed as a phagocytosis index.

### 2.10. Statistical Analysis 

The data are expressed as the mean and standard deviation (SD) of three replicates. The significance between stimulation and treatment was calculated using the non-parametric Mann–Whitney test or the *t*-test when appropriate. Statistical analysis was performed with GraphPad Prism^®^ 8 software. *p*-values less than 0.05 were considered significant.

## 3. Results

### 3.1. The Effect of PD and RES on Cell Viability, Death and Apoptosis Induced by Crystals

Preliminary experiments were conducted to assess cell toxicity of crystals and polyphenols. The MTT test showed a decrease in cell viability induced by CPP and MSU crystals which was abolished by PD and RES (supplementary material, Figure S1). 

Cell stimulation with CPP and MSU crystals caused an increase in cell death (1.7- and 2.3-fold over the basal condition, respectively) and apoptosis (1.6- and 2-fold, respectively). Both these effects were suppressed by PD and RES (supplementary material, Figures S2 and S3).

### 3.2. The Effect of PD and RES on Intra- and Extra-cellular Levels of IL-1β

**Figure 1:**
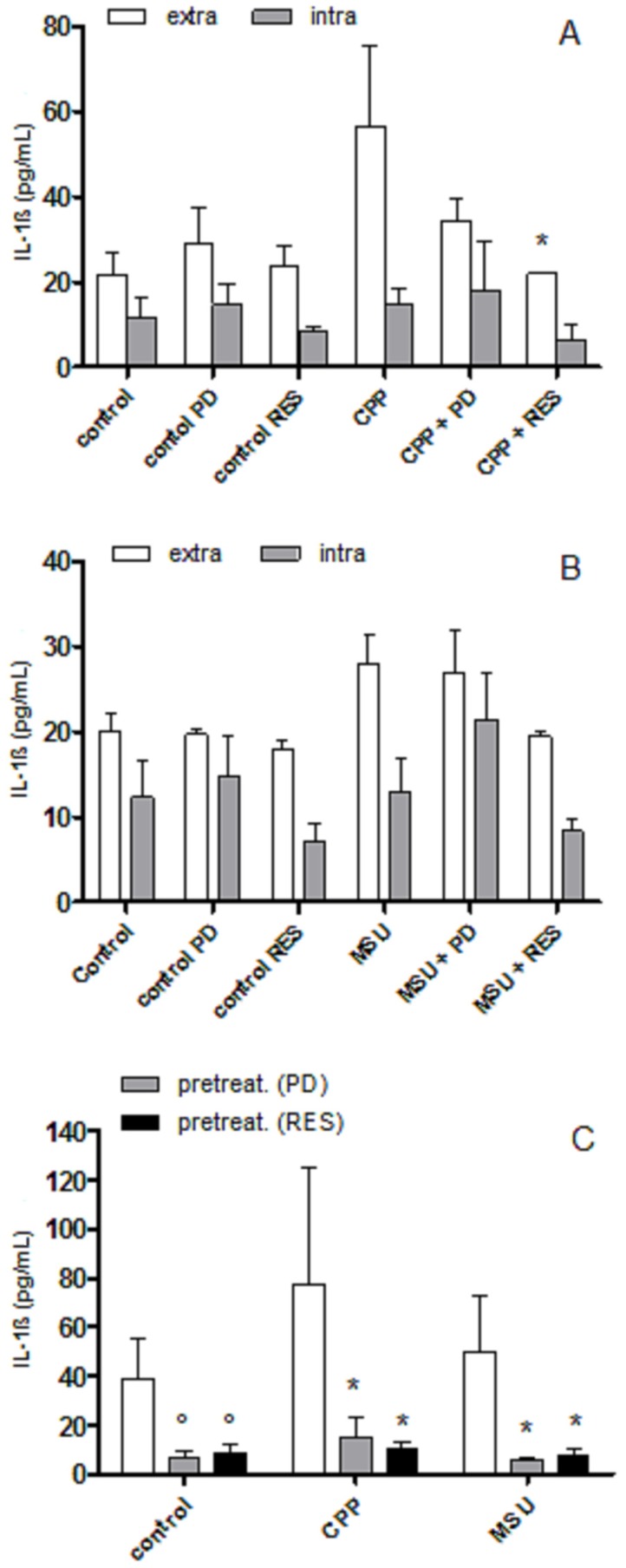
The effects of precursor polydatin (PD) and resveratol (RES) on calcium pyrophosphate (CPP) and monosodium urate (MSU) crystal-stimulated IL-1β production. Panel A, B: the cells were primed with PMA 100 ng/mL for 3 h and left overnight in fresh medium. The cells were then treated with CPP 0.025 mg/mL (**A**) or MSU 0.05 mg/mL (**B**) for 24 h in the presence of the two polyphenols. White bars show IL-1β extracellular concentrations, grey bars show IL-1β intracellular levels. In the left side of A and B figures are the control levels of IL-1β for each condition. Panel C: White bars show extracellular IL-1β level after crystal stimulation while grey and black bars show cytokine levels after 2 h cell pretreatment with PD (grey) and RES (black) followed by crystal stimulation. * *p* < 0.05 vs. CPP or MSU; ° *p* < 0.05 vs. basal control.

As shown in Figure 1 (panel A, B), RES inhibited extracellular levels of IL-1β induced by CPP (*p* < 0.05) and MSU crystals (non-significant) after 24 h, while PD showed an anti-inflammatory effect towards CPP only. Only RES was able to reduce IL-1β at the intra-cellular level. To evaluate whether the effect of PD and RES was mediated by a direct action on cell, THP-1 cells were pretreated with polyphenols and washed before crystal stimulation. Both PD and RES markedly reduced cytokine levels on culture medium in pretreated cells (Figure 1, panel C).

### 3.3. The Effect of PD and RES on IL-1β, NLRP3 and ASC Expression

Gene expression levels of IL-1β, an NLRP3 inflammasome and ASC, one of the inflammasome components, was examined after treatment and pretreatment with RES and PD.

**Figure 2:**
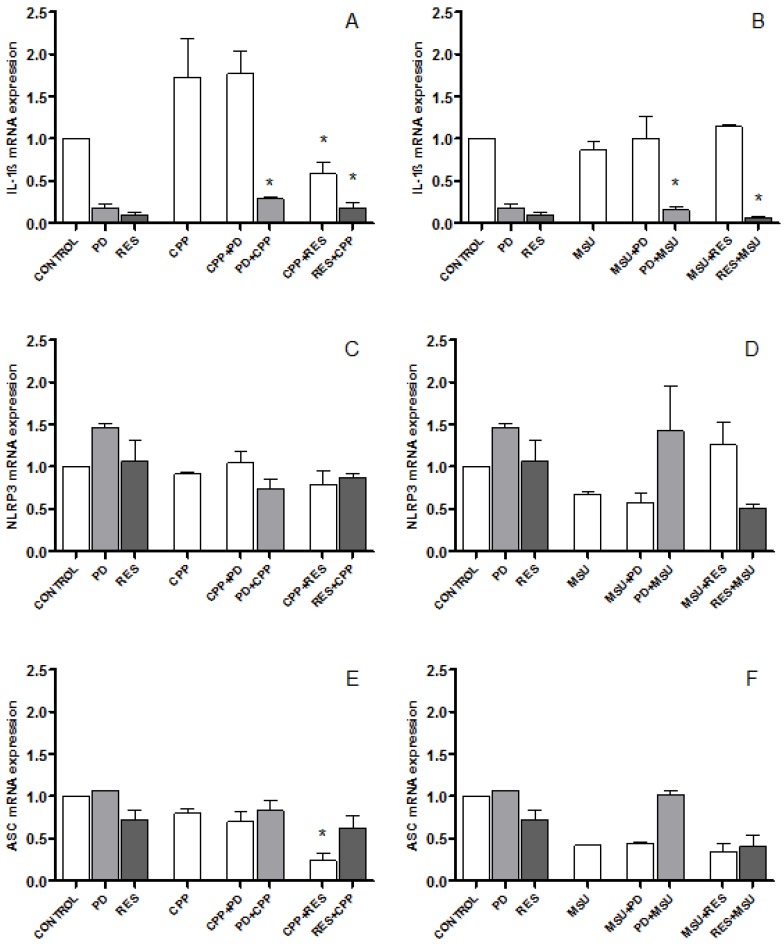
The effect of PD and RES on IL-1β, NLRP3 and ASC gene expression. Expression of IL-1β (**A**,**B**), NLRP3 (**C**,**D**) and ASC (**E**,**F**) mRNA in THP-1 cells stimulated with crystals and treated (white bars) or pretreated (gray bars) with PD and RES. * *p* < 0.05 vs. CPP or MSU.

As shown in Figure 2, CPP crystals induced a 1.7-fold change in IL-1β mRNA expression which was inhibited by RES only (panel A). By contrast, MSU crystals did not cause any change in IL-1β mRNA levels at the concentrations used in this study (panel B). The pretreatment of cells with PD and RES lead to a significant reduction of IL-1ß expression which was evident also at the basal conditions (panel A and B).

There was, instead, no significant effect on NLRP3 and ASC gene expression by both crystals and polyphenols, although RES decreased ASC gene expression after stimulation with CPP crystals (Figure 2, panel C–F).

### 3.4. RES and PD Inhibit ROS Production Induced by Crystals

**Figure 3:**
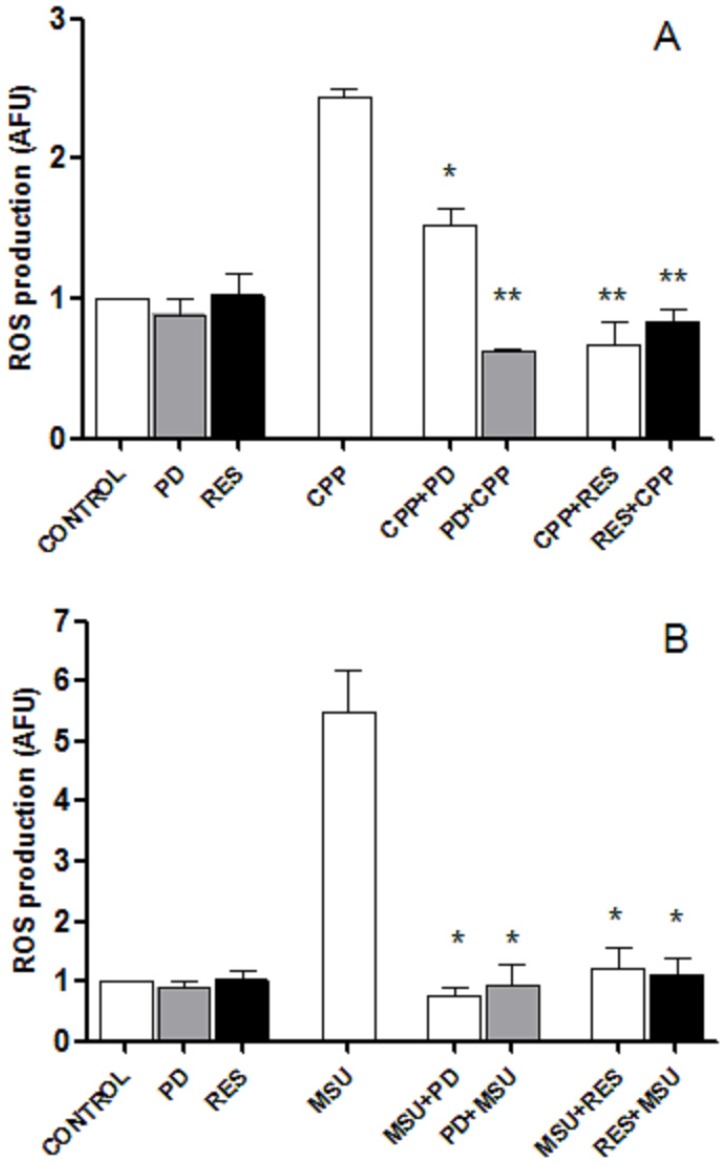
RES and PD suppress ROS production. White columns show ROS released by THP-1 cells treated with 0.025 mg/mL CPP (**A**) or 0.05 mg/mL MSU (**B**) crystals in presence of RES and PD. Grey and black columns indicate the amount of ROS released in cultures pretreated for 2 h with PD or RES, and treated with crystals after 24 h. On the left of the figures are the control conditions; CONTROL: cells only; PD: cells with PD only; RES, cells with RES only. Values are expressed as the mean ± standard deviation.* *p* < 0.05 vs. MSU or CPP.* *p* < 0.05, ** *p* < 0.01 vs. CPP or MSU.

As ROS production has been demonstrated to play an important role in crystal-induced inflammation, the influence of the polyphenols on ROS released from treated and pretreated cells was investigated. THP-1 cells stimulated with CPP and MSU crystals for 24 h increased ROS production by approximately 2.4- and 5.5-fold compared with their basal level. Both RES and PD were significantly effective in inhibiting ROS production when added along with the crystals or 2 h before (Figure 3). 

### 3.5. RES and PD Inhibit Nitric oxide Production Induced by Crystals

**Figure 4:**
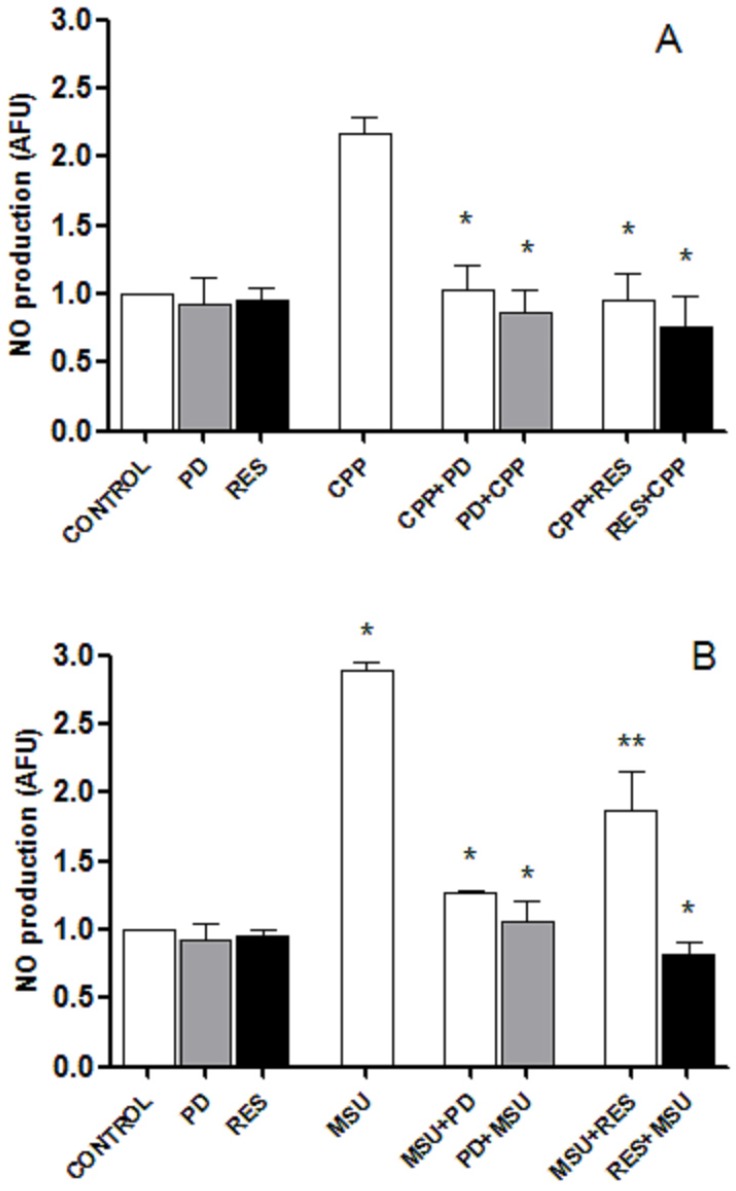
RES and PD decrease NO production. White columns show quantification of NO production by THP-1 cells treated with 0.025 mg/mL CPP (**A**) or 0.05 mg/mL MSU (**B**) crystals in presence of RES and PD. Grey and black columns indicate the amount of NO released in cultures pretreated for 2 h with PD or RES, and treated with crystals after 24 h. On the left of the figures are the control conditions; CONTROL: cells only; PD: cells with PD only; RES, cells with RES only. Values are expressed as the mean ± standard deviation.* *p* < 0.01 vs. MSU or CPP; ** *p* < 0.05 vs. MSU.

CPP and MSU crystals induced a significant, although moderate, release of NO after 24 h stimulation with respect to control (Figure 4). In the presence of RES and PD, the amount of NO returned to basal levels. Similarly, the pretreatment of cells with the two polyphenols inhibited the production of NO in culture supernatants.

### 3.6. The Effect of RES and PD on Crystal Phagocytosis

As crystal phagocytosis represents a fundamental step in triggering the inflammatory response to crystals, the hypothesis that PD and RES could interfere with crystal internalization has been examined. 

**Figure 5:**
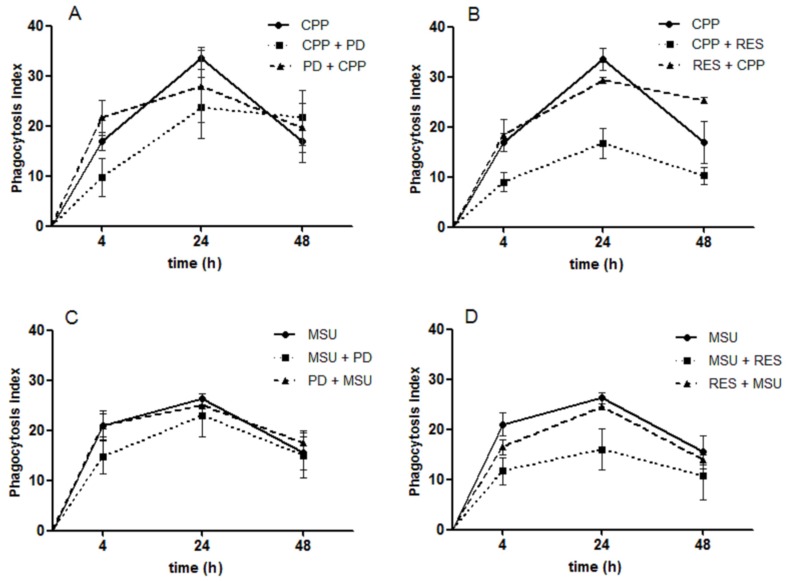
The effect of PD and RES on CPP and MSU crystal phagocytosis. (**A**) cells stimulated with CPP crystals and treated (square) or pretreated (triangle) with PD; (**B**) cells stimulated with CPP crystals and treated (square) or pretreated (triangle) with RES; (**C**) cells stimulated with MSU crystals and treated (square) or pretreated (triangle) with PD; (**D**) cells stimulated with MSU crystals and treated (square) or pretreated (triangle) with RES. Phagocytosis was evaluated using ordinary/polarized light microscopy assessing the presence of intracellular crystals at the indicated time points. Crystals were used at the concentration of 0.025 (CPP) and 0.05 mg/mL (MSU). * *p* < 0.05 vs. CPP.

The phagocytosis was measured at different time points (4, 24, and 48 h) on cells treated or pretreated with PD and RES. Figure 5 shows the variation of the phagocytosis index over time. While PD reduced in a non-significant manner the uptake of crystals, the phagocytosis index diminished significantly in presence of RES. However, the pretreatment of the cells with the two polyphenols had no effect on the phagocytosis process.

## 4. Discussion

This study showed that RES and PD, two important polyphenols found in grape juice and red wine [24], reduce IL-1β release in THP-1 cells stimulated by pathogenic crystals affecting inflammasome activity but not its gene expression. We have found that these compounds influence the production of ROS and NO and show a strong preventing effect towards the release of these inflammatory mediators. We also demonstrated that RES had a stronger anti-inflammatory effect with respect to PD, explained, at least in part, by its capacity to inhibit the process of crystal phagocytosis.

In humans, CPP and MSU crystal deposition triggers powerful inflammatory reactions causing, respectively, calcium pyrophosphate crystal-associated arthritis (pseudogout) and gout. Both types of crystals activate specific signaling pathways leading to the production of inflammatory cytokines, chemokines, and pro-oxidant molecules in the synovial compartment [25]. As stated above, IL-1β is one of the most important mediators in crystal-induced inflammation [3,5].

It is known that a co-stimulus is needed to prime cells to generate IL-1β when exposed to pathogenic crystals. It has been hypothesized that serum proteins [26,27] and Toll-like receptor ligands such as free fatty acids [28] synergize with crystals on IL-1β induction. In this study, we used the protein kinase C activator PMA which influences cell differentiation and stimulates monocyte functions. Using this model, we previously demonstrated that the most important catechin of green tea, epigallocatechin-gallate, leads to a significant inhibition of the inflammatory response triggered by CPP crystals [12]. In the present study, we observed an increase of IL-1β production induced by crystals after cell priming, which was inhibited by RES and PD both at intracellular and extracellular levels. The inflammatory effect of CPP in this cellular model was more pronounced with respect to that induced by MSU crystals. 

CPP, but not MSU, crystals induced an increase in IL-1β mRNA levels which was inhibited only by RES. By contrast, the pretreatment of cells with PD and RES caused a significant reduction of IL-1β gene expression which was evident also at the basal conditions. There was, instead, no significant effect on NLRP3 and ASC gene expression by both crystals and polyphenols, although RES diminished ASC gene expression after treatment with CPP crystals. Therefore, the results showed that the decrease in IL-1β observed in THP-1 cells stimulated with crystals and treated with RES and PD was associated with a decrease in IL-1β gene expression but not in NLRP3 and ASC gene expression.

Recently, Yang et al. showed that RES decreases IL-1β levels in peripheral blood mononuclear cells obtained from patients with gout and challenged with MSU crystals. According to their results, RES was able to restore the levels of sirtuin-1 protein previously decreased by crystals [29]. In that study, RES showed opposite effects on pro-IL-1β and the mature form, depending on the concentration of the polyphenol. 

RES, along with red wine extracts, has been shown to decrease IL-1β secretion in murine macrophage prior to cell priming with LPS [30]. In particular, the authors showed that RES was able to decrease IL-1β production and NLRP3 expression after pretreatment, and demonstrated that the effect of this polyphenol on the IL-1 activation pathway can differ considerably depending on the NLRP3 activator used. 

In line with that study, our results showed that the pretreatment of cells with the two polyphenols almost completely abrogated the inflammatory response, suggesting a major role on cell priming rather than on the activation signal. Of note, PD and RES were removed from the cultures after a 2 h pretreatment, suggesting a sustained anti-inflammatory effect over time. Cell pretreatment conditioned the basal state of the cells, making them less prone to stimulation.

We then investigated whether RES and PD were able to affect oxidative and nitrosative stress which has been demonstrated to be induced by crystals in synoviocytes, chondrocytes, and macrophages [20,31,32]. In our experiments, RES and PD decreased ROS and NO production, and the effect was more effective when cells were pretreated with the two compounds. 

These effects could be related to the affinity of these compounds for the lipid part of membranes interacting with the head groups of phospholipids [33]. This mechanism has been postulated to protect lipid membranes from oxidative stress and hydrolytic attack [33].

The importance of this lipid protective effect has recently emerged by using PD as topical applications to prevent drug-induced skin toxicities [34]. 

We finally investigated whether PD and RES could interfere with crystal phagocytosis, which represents a fundamental step in triggering the inflammatory response to crystals. We observed that RES significantly inhibited CPP and MSU crystal internalization when added concurrently with the crystals but they had no effect after cell pretreatment. The influence of RES on phagocytosis has been described in different cellular models. It has been demonstrated to inhibit bacteria phagocytosis in THP-1 cells [35] and the phagocytic activity of microglia [36] as well as to enhance the phagocytosis of yeast in human macrophage-like cells [37]. In THP-1 cells, this effect has been observed to be mediated by gene expression inhibition of the phagocytic scavenger receptors and C-type lectin receptors carried out by RES [35]. 

In our conditions, it could be hypothesized that RES acts on innate immunity surface receptors, such as TLRs, whose ligands serve to prime cells in the signaling transduction pathway activated by crystals. However, TLR-2 and TLR-4 inhibition by monoclonal antibodies does not affect the rate of CPP and MSU crystals phagocytosis in this model (personal experimental observations). Whether RES acts on other types of receptors will be investigated in further studies.

As far as PD is concerned, it showed only a modest effect on phagocytosis. With respect to RES, PD has a glucoside group which seems to confer to the molecule’s different biological properties [38]. Among these, it has been demonstrated that PD, unlike RES which penetrates the cell passively, enters the cell via an active mechanism using glucose carriers [39]. 

In our model, PD showed a smaller effect on IL-1 inhibition compared to RES but a strong anti-oxidant effect towards the release of ROS and NO. 

A limitation of this study might concern the concentration of RES used in this study, which, although in line with other in vitro studies, is probably too high to be reached in the bloodstream after dietary RES consumption. However, it is possible to achieve such plasmatic concentration by administering resveratrol supplements [40]. Furthermore, the conjugated glucuronides and sulfate metabolites of RES retain biological activity [41].

## 5. Conclusions

In conclusion, our study demonstrated that PD, and to a major extent RES, have a strong preventive anti-inflammatory effect in THP-1 cells primed with PMA and stimulated with pathogenic crystals. Whether their mechanism of action is related to the interaction of these compounds with cell membranes and to the protection of cells from oxidative reactions, might represent a future focus of research.

Finally, given the auto-inflammatory nature of crystal-induced arthritis with acute attacks which resolve spontaneously, dietary supplementation with these polyphenols could support pharmacological treatment in patients and prevent the acute phase of the disease. Clinical studies assessing the benefits of RES and PD in the management of gout and pseudogout as well as in reducing biological inflammatory biomarkers could provide further data on the anti-inflammatory preventive effect of these polyphenols and their possible application in those diseases. 

## Figures and Tables

**Table 1:** Primer sets used for quantitative real-time PCR.

RNA Template	Forward Primer (5′-3′)	Reverse Primer (5′-3′)
GAPDH	CAAAGTTGTCATGGATGACC	CCATGGAGAAGGCTGGGG
IL-1β	CTGTCCTGCGTGTTGAAAGA	TTGGGTAATTTTTGGGATCTACA
ASC	CTGTCCATGGACGCCTTGG	CATCCGTCAGGACCTTCCCGT
NLRP3	CACCTGTTGTGCAATCTGAAG	GCAAGATCCTGACAACATGC

## Supplementary Materials

The following are available online at https://www.mdpi.com/2304-8158/8/11/560/s1, Figure S1. The effect of PD and RES on cell viability induced by crystals, Figure S2. The effect of PD and RES on cell death induced by crystals, Figure S3. The effect of PD and RES on cell apoptosis induced by crystals.
