# Supplementary material for: Polydatin and Resveratrol Inhibit the Inflammatory Process Induced by Urate and Pyrophosphate Crystals in THP-1 Cells
Source: Foods. 2019 Nov 7;8(11):560. doi: 10.3390/foods8110560 (PMC6915461; doi:10.3390/foods8110560)
Supplement: Supplementary file 1 [file foods-08-00560-s001.pdf]

Article

Polydatin and resveratrol inhibit the inflammatory process induced by urate and pyrophosphate crystals in THP-1 cells.

Francesca Oliviero[1*#], Yessica Zamudio-Cuevas[1,2#], Elisa Belluzzi [1], Lisa Andretto [1], Anna Scanu[1], Marta Favero[1], Roberta Ramonda[1], Giampietro Ravagnan[3], Alberto López-Reyes[2], Paolo Spinella[4], Leonardo Punzi[1,5].

SUPPLEMENTARY MATERIALS

Figure S1. The effect of PD and RES on cell viability induced by crystals.

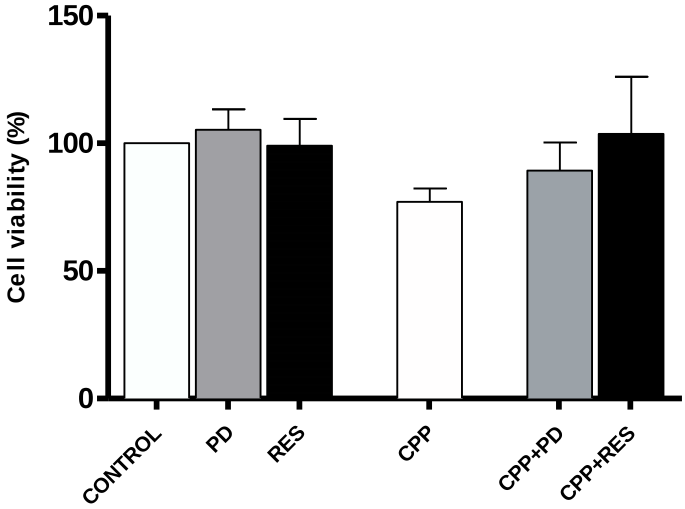

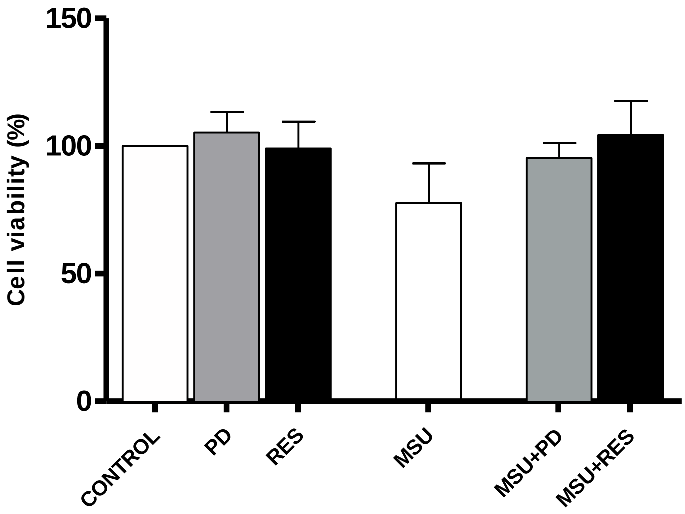

White columns show cell viability percentage in THP-1 cells (control) and in THP-1 cells treated with CPP or MSU crystals. Grey and black column indicate the amount of cell viability in cultures stimulated with crystals and treated with PD or RES, respectively. Values are expressed as the mean ± standard deviation.

Figure S2. The effect of PD and RES on cell death induced by crystals.

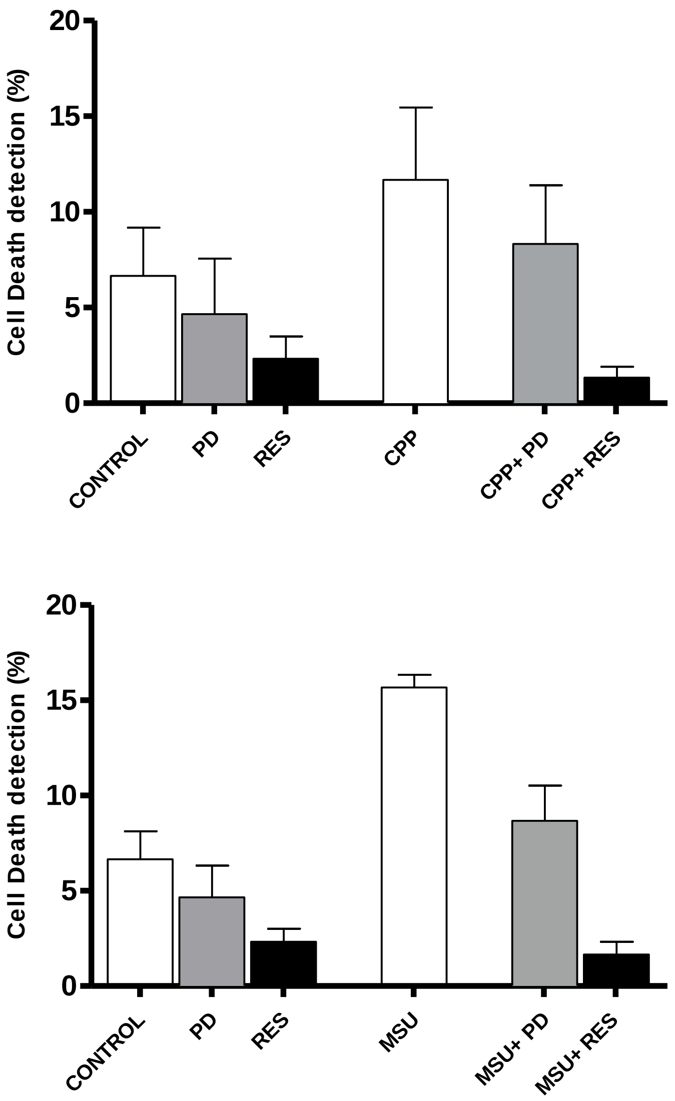

White columns show cell death percentage in THP-1 cells (control) and in THP-1 cells treated with CPP or MSU crystals. Grey and black column indicate the amount of cell death in cultures stimulated with crystals and treated with PD or RES, respectively. Values are expressed as the mean ± standard deviation.

Figure S3. The effect of PD and RES on cell apoptosis induced by crystals.

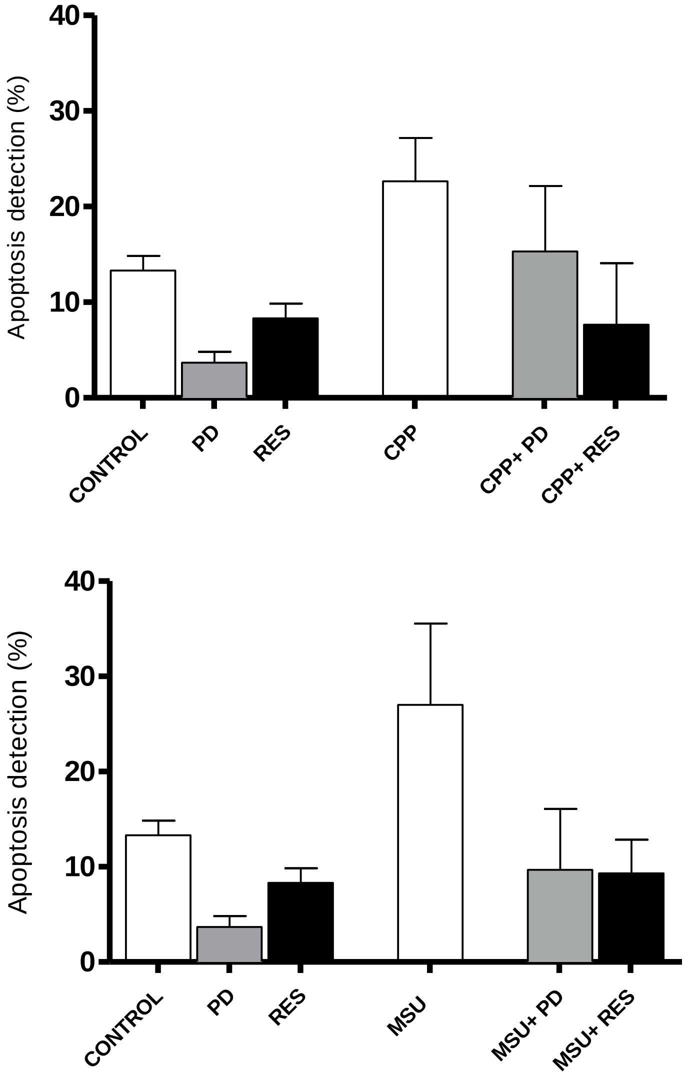

White columns show cell apoptosis percentage in THP-1 cells (control) and in THP-1 cells treated with CPP or MSU crystals. Grey and black column indicate the amount of apoptosis in cultures stimulated with crystals and treated with PD or RES, respectively. Values are expressed as the mean ± standard deviation.
